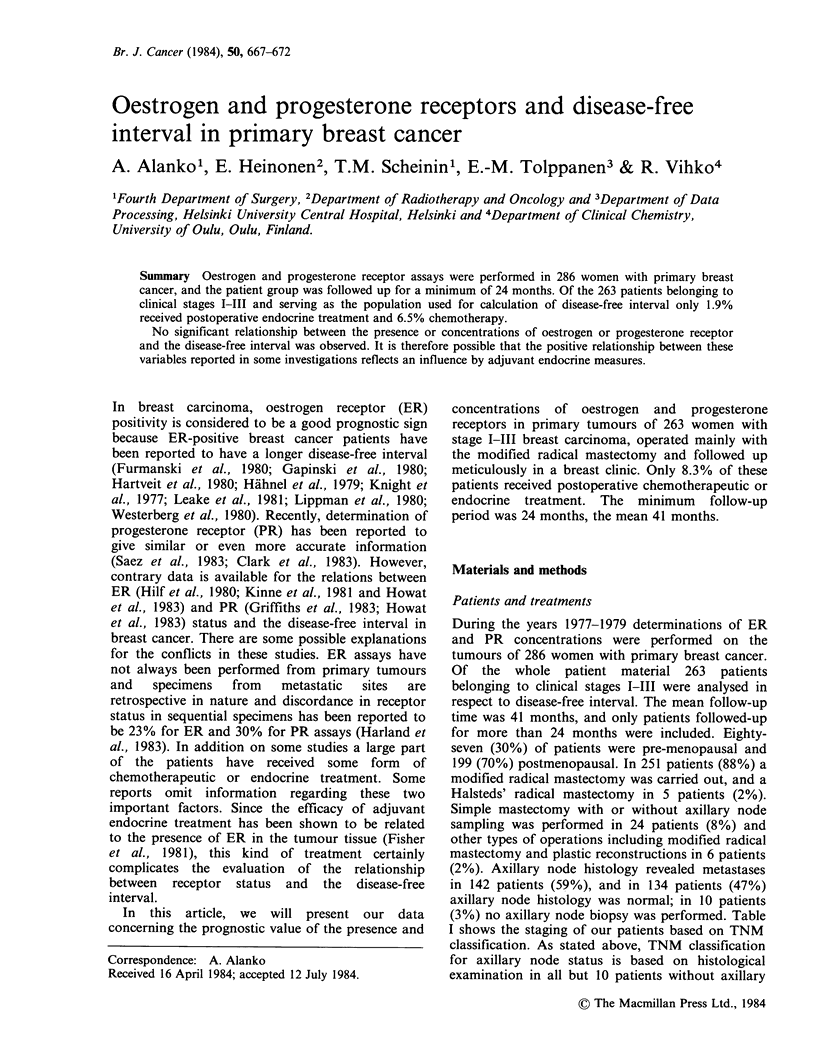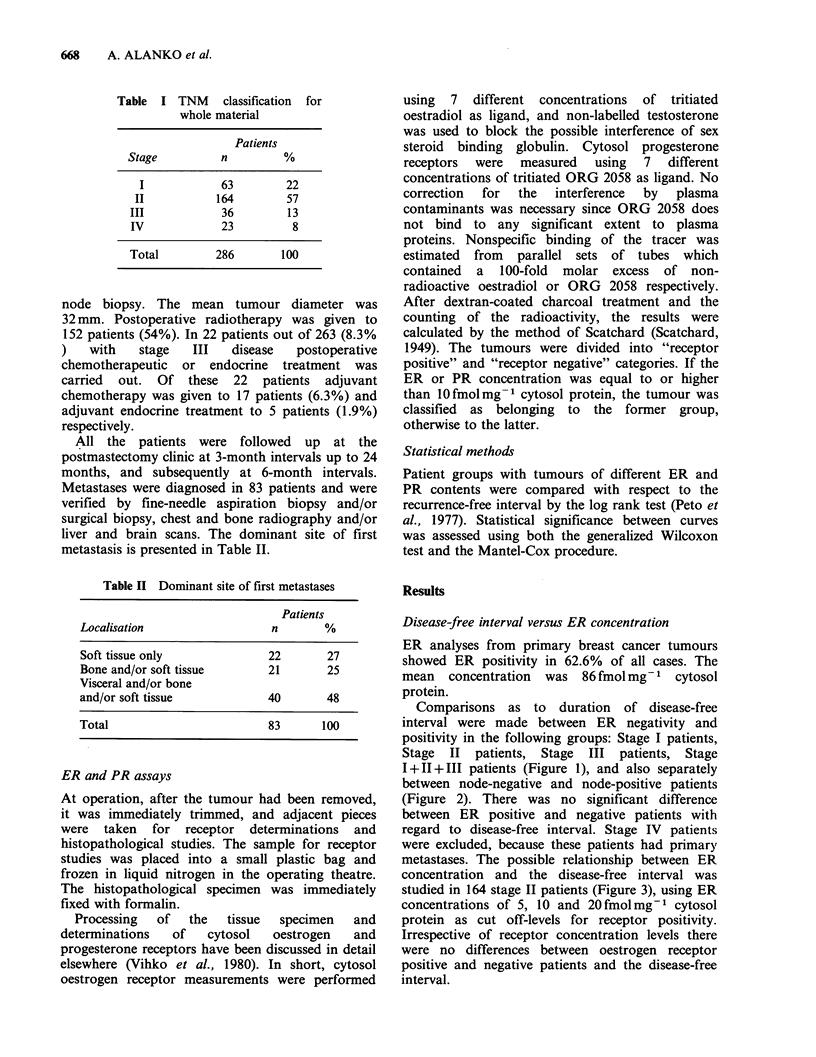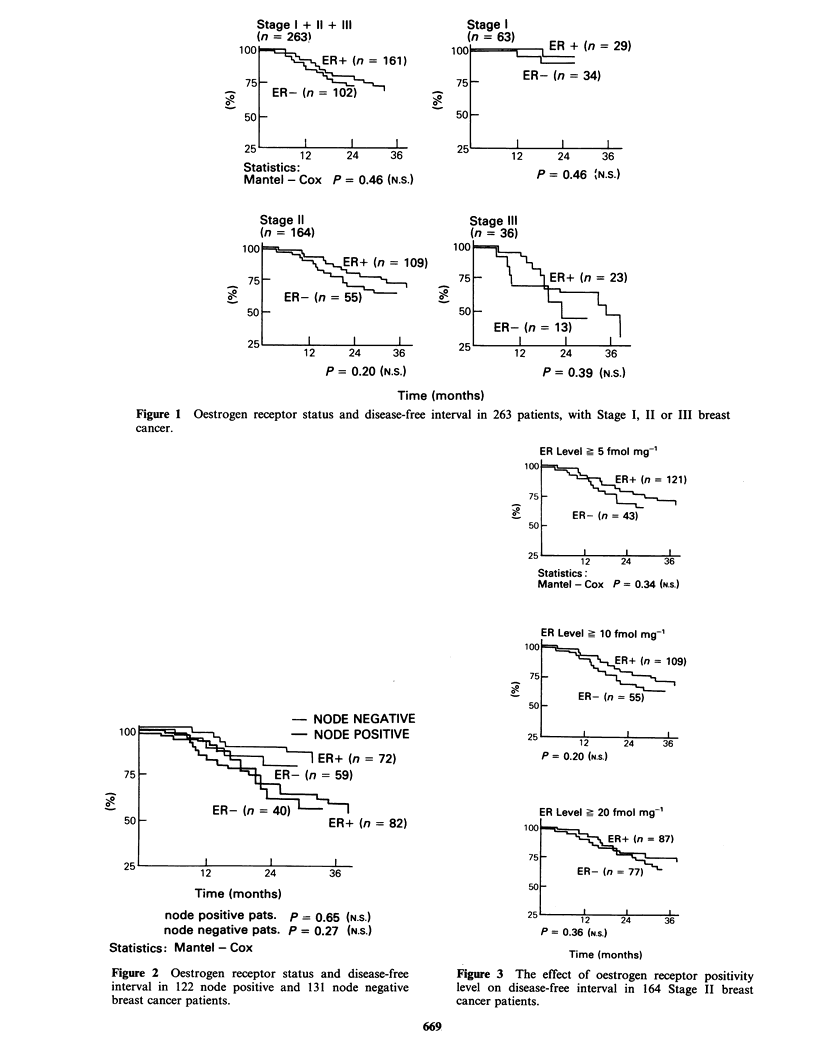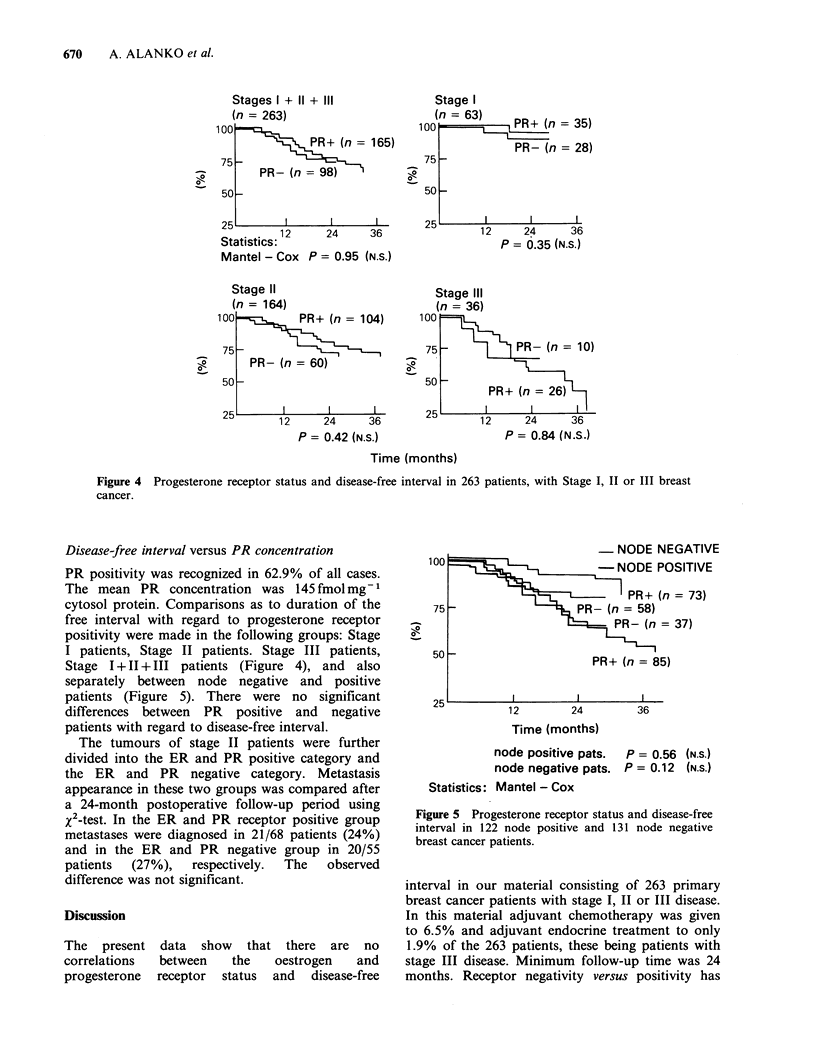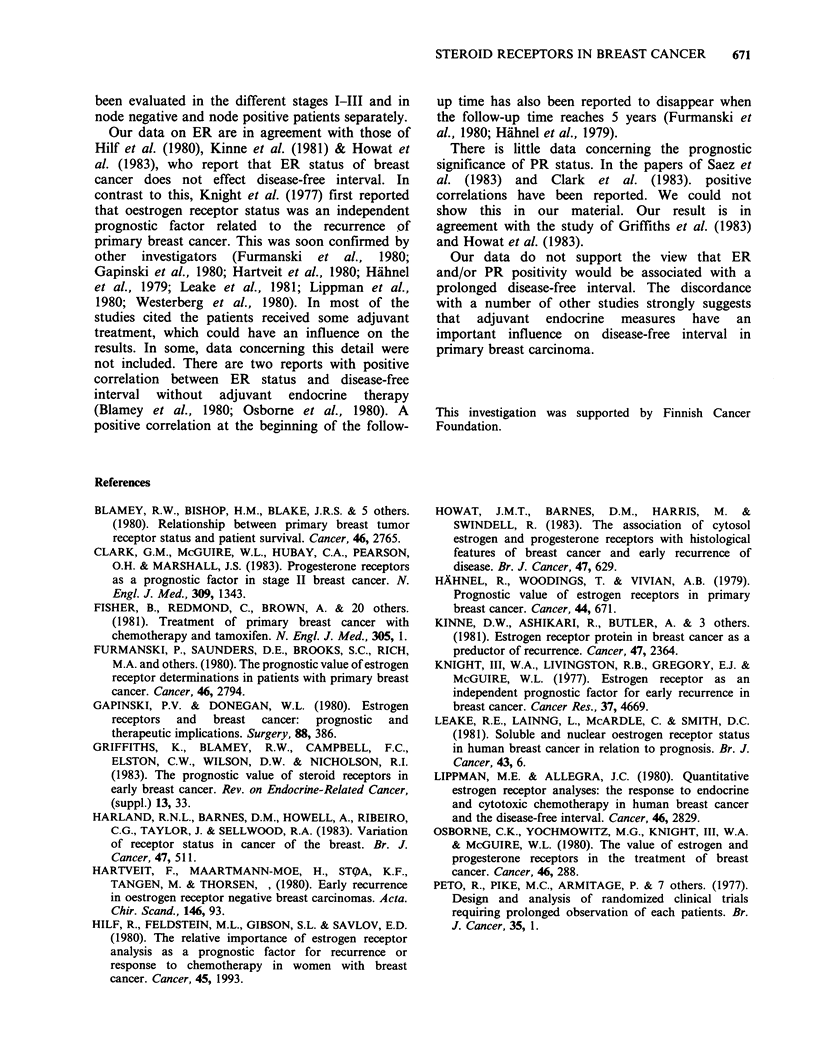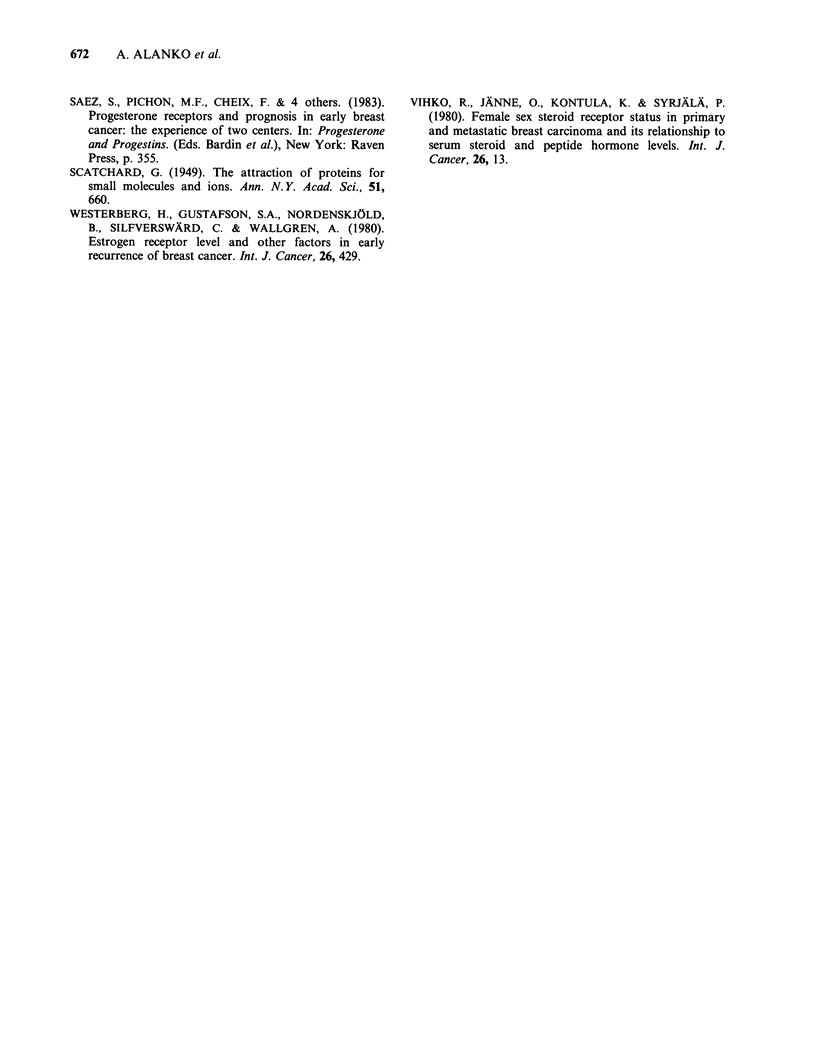# Oestrogen and progesterone receptors and disease-free interval in primary breast cancer.

**DOI:** 10.1038/bjc.1984.234

**Published:** 1984-11

**Authors:** A. Alanko, E. Heinonen, T. M. Scheinin, E. M. Tolppanen, R. Vihko

## Abstract

Oestrogen and progesterone receptor assays were performed in 286 women with primary breast cancer, and the patient group was followed up for a minimum of 24 months. Of the 263 patients belonging to clinical stages I-III and serving as the population used for calculation of disease-free interval only 1.9% received postoperative endocrine treatment and 6.5% chemotherapy. No significant relationship between the presence or concentrations of oestrogen or progesterone receptor and the disease-free interval was observed. It is therefore possible that the positive relationship between these variables reported in some investigations reflects an influence by adjuvant endocrine measures.


*Br. J. Cancer* (1984), **50**, 667–672

### Oestrogen and progesterone receptors and disease-free interval in primary breast cancer

A. Alanko[1], E. Heinonen[2], T.M. Scheinin[1], E.-M. Tolppanen[3] & R. Vihko[4]

[1]*Fourth Department of Surgery,* [2]*Department of Radiotherapy and Oncology and* [3]*Department of Data Processing, Helsinki University Central Hospital, Helsinki and* [4]*Department of Clinical Chemistry, University of Oulu, Oulu, Finland.*

**Summary** Oestrogen and progesterone receptor assays were performed in 286 women with primary breast cancer, and the patient group was followed up for a minimum of 24 months. Of the 263 patients belonging to clinical stages I–III and serving as the population used for calculation of disease-free interval only 1.9% received postoperative endocrine treatment and 6.5% chemotherapy.

No significant relationship between the presence or concentrations of oestrogen or progesterone receptor and the disease-free interval was observed. It is therefore possible that the positive relationship between these variables reported in some investigations reflects an influence by adjuvant endocrine measures.

In breast carcinoma, oestrogen receptor (ER) positivity is considered to be a good prognostic sign because ER-positive breast cancer patients have been reported to have a longer disease-free interval (Furmanski *et al.*, 1980; Gapinski *et al.*, 1980; Hartveit *et al.*, 1980; Hähnel *et al.*, 1979; Knight *et al.*, 1977; Leake *et al.*, 1981; Lippman *et al.*, 1980; Westerberg *et al.*, 1980). Recently, determination of progesterone receptor (PR) has been reported to give similar or even more accurate information (Saez *et al.*, 1983; Clark *et al.*, 1983). However, contrary data is available for the relations between ER (Hilf *et al.*, 1980; Kinne *et al.*, 1981 and Howat *et al.*, 1983) and PR (Griffiths *et al.*, 1983; Howat *et al.*, 1983) status and the disease-free interval in breast cancer. There are some possible explanations for the conflicts in these studies. ER assays have not always been performed from primary tumours and specimens from metastatic sites are retrospective in nature and discordance in receptor status in sequential specimens has been reported to be 23% for ER and 30% for PR assays (Harland *et al.*, 1983). In addition on some studies a large part of the patients have received some form of chemotherapeutic or endocrine treatment. Some reports omit information regarding these two important factors. Since the efficacy of adjuvant endocrine treatment has been shown to be related to the presence of ER in the tumour tissue (Fisher *et al.*, 1981), this kind of treatment certainly complicates the evaluation of the relationship between receptor status and the disease-free interval.

In this article, we will present our data concerning the prognostic value of the presence and concentrations of oestrogen and progesterone receptors in primary tumours of 263 women with stage I–III breast carcinoma, operated mainly with the modified radical mastectomy and followed up meticulously in a breast clinic. Only 8.3% of these patients received postoperative chemotherapeutic or endocrine treatment. The minimum follow-up period was 24 months, the mean 41 months.

Correspondence: A. Alanko
Received 16 April 1984; accepted 12 July 1984.

### Materials and methods

### *Patients and treatments*

During the years 1977–1979 determinations of ER and PR concentrations were performed on the tumours of 286 women with primary breast cancer. Of the whole patient material 263 patients belonging to clinical stages I–III were analysed in respect to disease-free interval. The mean follow-up time was 41 months, and only patients followed-up for more than 24 months were included. Eighty-seven (30%) of patients were pre-menopausal and 199 (70%) postmenopausal. In 251 patients (88%) a modified radical mastectomy was carried out, and a Halsteds' radical mastectomy in 5 patients (2%). Simple mastectomy with or without axillary node sampling was performed in 24 patients (8%) and other types of operations including modified radical mastectomy and plastic reconstructions in 6 patients (2%). Axillary node histology revealed metastases in 142 patients (59%), and in 134 patients (47%) axillary node histology was normal; in 10 patients (3%) no axillary node biopsy was performed. Table I shows the staging of our patients based on TNM classification. As stated above, TNM classification for axillary node status is based on histological examination in all but 10 patients without axillary

© The Macmillan Press Ltd., 1984

**Table I** TNM classification for whole material

| Stage | Patients n | % |
|---|---|---|
| I | 63 | 22 |
| II | 164 | 57 |
| III | 36 | 13 |
| IV | 23 | 8 |
| Total | 286 | 100 |

node biopsy. The mean tumour diameter was 32 mm. Postoperative radiotherapy was given to 152 patients (54%). In 22 patients out of 263 (8.3% ) with stage III disease postoperative chemotherapeutic or endocrine treatment was carried out. Of these 22 patients adjuvant chemotherapy was given to 17 patients (6.3%) and adjuvant endocrine treatment to 5 patients (1.9%) respectively.

All the patients were followed up at the postmastectomy clinic at 3-month intervals up to 24 months, and subsequently at 6-month intervals. Metastases were diagnosed in 83 patients and were verified by fine-needle aspiration biopsy and/or surgical biopsy, chest and bone radiography and/or liver and brain scans. The dominant site of first metastasis is presented in Table II.

**Table II** Dominant site of first metastases

| Localisation | Patients n | % |
|---|---|---|
| Soft tissue only | 22 | 27 |
| Bone and/or soft tissue | 21 | 25 |
| Visceral and/or bone and/or soft tissue | 40 | 48 |
| Total | 83 | 100 |

*ER and PR assays*

At operation, after the tumour had been removed, it was immediately trimmed, and adjacent pieces were taken for receptor determinations and histopathological studies. The sample for receptor studies was placed into a small plastic bag and frozen in liquid nitrogen in the operating theatre. The histopathological specimen was immediately fixed with formalin.

Processing of the tissue specimen and determinations of cytosol oestrogen and progesterone receptors have been discussed in detail elsewhere (Vihko *et al.*, 1980). In short, cytosol oestrogen receptor measurements were performed using 7 different concentrations of tritiated oestradiol as ligand, and non-labelled testosterone was used to block the possible interference of sex steroid binding globulin. Cytosol progesterone receptors were measured using 7 different concentrations of tritiated ORG 2058 as ligand. No correction for the interference by plasma contaminants was necessary since ORG 2058 does not bind to any significant extent to plasma proteins. Nonspecific binding of the tracer was estimated from parallel sets of tubes which contained a 100-fold molar excess of non-radioactive oestradiol or ORG 2058 respectively. After dextran-coated charcoal treatment and the counting of the radioactivity, the results were calculated by the method of Scatchard (Scatchard, 1949). The tumours were divided into "receptor positive" and "receptor negative" categories. If the ER or PR concentration was equal to or higher than 10 $fmol\,mg^{-1}$ cytosol protein, the tumour was classified as belonging to the former group, otherwise to the latter.

*Statistical methods*

Patient groups with tumours of different ER and PR contents were compared with respect to the recurrence-free interval by the log rank test (Peto *et al.*, 1977). Statistical significance between curves was assessed using both the generalized Wilcoxon test and the Mantel-Cox procedure.

### Results

*Disease-free interval versus ER concentration*

ER analyses from primary breast cancer tumours showed ER positivity in 62.6% of all cases. The mean concentration was 86 $fmol\,mg^{-1}$ cytosol protein.

Comparisons as to duration of disease-free interval were made between ER negativity and positivity in the following groups: Stage I patients, Stage II patients, Stage III patients, Stage I+II+III patients (Figure 1), and also separately between node-negative and node-positive patients (Figure 2). There was no significant difference between ER positive and negative patients with regard to disease-free interval. Stage IV patients were excluded, because these patients had primary metastases. The possible relationship between ER concentration and the disease-free interval was studied in 164 stage II patients (Figure 3), using ER concentrations of 5, 10 and 20 $fmol\,mg^{-1}$ cytosol protein as cut off-levels for receptor positivity. Irrespective of receptor concentration levels there were no differences between oestrogen receptor positive and negative patients and the disease-free interval.

**Figure 1** Oestrogen receptor status and disease-free interval in 263 patients, with Stage I, II or III breast cancer.

**Figure 2** Oestrogen receptor status and disease-free interval in 122 node positive and 131 node negative breast cancer patients.

**Figure 3** The effect of oestrogen receptor positivity level on disease-free interval in 164 Stage II breast cancer patients.

**Figure 4** Progesterone receptor status and disease-free interval in 263 patients, with Stage I, II or III breast cancer.

*Disease-free interval* versus *PR concentration*

PR positivity was recognized in 62.9% of all cases. The mean PR concentration was 145 fmol mg$^{-1}$ cytosol protein. Comparisons as to duration of the free interval with regard to progesterone receptor positivity were made in the following groups: Stage I patients, Stage II patients. Stage III patients, Stage I+II+III patients (Figure 4), and also separately between node negative and positive patients (Figure 5). There were no significant differences between PR positive and negative patients with regard to disease-free interval.

The tumours of stage II patients were further divided into the ER and PR positive category and the ER and PR negative category. Metastasis appearance in these two groups was compared after a 24-month postoperative follow-up period using $\chi^2$-test. In the ER and PR receptor positive group metastases were diagnosed in 21/68 patients (24%) and in the ER and PR negative group in 20/55 patients (27%), respectively. The observed difference was not significant.

**Figure 5** Progesterone receptor status and disease-free interval in 122 node positive and 131 node negative breast cancer patients.

### Discussion

The present data show that there are no correlations between the oestrogen and progesterone receptor status and disease-free interval in our material consisting of 263 primary breast cancer patients with stage I, II or III disease. In this material adjuvant chemotherapy was given to 6.5% and adjuvant endocrine treatment to only 1.9% of the 263 patients, these being patients with stage III disease. Minimum follow-up time was 24 months. Receptor negativity *versus* positivity has been evaluated in the different stages I–III and in node negative and node positive patients separately.

Our data on ER are in agreement with those of Hilf *et al.* (1980), Kinne *et al.* (1981) & Howat *et al.* (1983), who report that ER status of breast cancer does not effect disease-free interval. In contrast to this, Knight *et al.* (1977) first reported that oestrogen receptor status was an independent prognostic factor related to the recurrence of primary breast cancer. This was soon confirmed by other investigators (Furmanski *et al.*, 1980; Gapinski *et al.*, 1980; Hartveit *et al.*, 1980; Hähnel *et al.*, 1979; Leake *et al.*, 1981; Lippman *et al.*, 1980; Westerberg *et al.*, 1980). In most of the studies cited the patients received some adjuvant treatment, which could have an influence on the results. In some, data concerning this detail were not included. There are two reports with positive correlation between ER status and disease-free interval without adjuvant endocrine therapy (Blamey *et al.*, 1980; Osborne *et al.*, 1980). A positive correlation at the beginning of the follow-up time has also been reported to disappear when the follow-up time reaches 5 years (Furmanski *et al.*, 1980; Hähnel *et al.*, 1979).

There is little data concerning the prognostic significance of PR status. In the papers of Saez *et al.* (1983) and Clark *et al.* (1983). positive correlations have been reported. We could not show this in our material. Our result is in agreement with the study of Griffiths *et al.* (1983) and Howat *et al.* (1983).

Our data do not support the view that ER and/or PR positivity would be associated with a prolonged disease-free interval. The discordance with a number of other studies strongly suggests that adjuvant endocrine measures have an important influence on disease-free interval in primary breast carcinoma.

This investigation was supported by Finnish Cancer Foundation.

### References

BLAMEY, R.W., BISHOP, H.M., BLAKE, J.R.S. & 5 others. (1980). Relationship between primary breast tumor receptor status and patient survival. *Cancer*, **46**, 2765.

CLARK, G.M., McGUIRE, W.L., HUBAY, C.A., PEARSON, O.H. & MARSHALL, J.S. (1983). Progesterone receptors as a prognostic factor in stage II breast cancer. *N. Engl. J. Med.*, **309**, 1343.

FISHER, B., REDMOND, C., BROWN, A. & 20 others. (1981). Treatment of primary breast cancer with chemotherapy and tamoxifen. *N. Engl. J. Med.*, **305**, 1.

FURMANSKI, P., SAUNDERS, D.E., BROOKS, S.C., RICH, M.A. and others. (1980). The prognostic value of estrogen receptor determinations in patients with primary breast cancer. *Cancer*, **46**, 2794.

GAPINSKI, P.V. & DONEGAN, W.L. (1980). Estrogen receptors and breast cancer: prognostic and therapeutic implications. *Surgery*, **88**, 386.

GRIFFITHS, K., BLAMEY, R.W., CAMPBELL, F.C., ELSTON, C.W., WILSON, D.W. & NICHOLSON, R.I. (1983). The prognostic value of steroid receptors in early breast cancer. *Rev. on Endocrine-Related Cancer*, (suppl.) **13**, 33.

HARLAND, R.N.L., BARNES, D.M., HOWELL, A., RIBEIRO, C.G., TAYLOR, J. & SELLWOOD, R.A. (1983). Variation of receptor status in cancer of the breast. *Br. J. Cancer*, **47**, 511.

HARTVEIT, F., MAARTMANN-MOE, H., STØA, K.F., TANGEN, M. & THORSEN, , (1980). Early recurrence in oestrogen receptor negative breast carcinomas. *Acta. Chir. Scand.*, **146**, 93.

HILF, R., FELDSTEIN, M.L., GIBSON, S.L. & SAVLOV, E.D. (1980). The relative importance of estrogen receptor analysis as a prognostic factor for recurrence or response to chemotherapy in women with breast cancer. *Cancer*, **45**, 1993.

HOWAT, J.M.T., BARNES, D.M., HARRIS, M. & SWINDELL, R. (1983). The association of cytosol estrogen and progesterone receptors with histological features of breast cancer and early recurrence of disease. *Br. J. Cancer*, **47**, 629.

HÄHNEL, R., WOODINGS, T. & VIVIAN, A.B. (1979). Prognostic value of estrogen receptors in primary breast cancer. *Cancer*, **44**, 671.

KINNE, D.W., ASHIKARI, R., BUTLER, A. & 3 others. (1981). Estrogen receptor protein in breast cancer as a preductor of recurrence. *Cancer*, **47**, 2364.

KNIGHT, III, W.A., LIVINGSTON, R.B., GREGORY, E.J. & McGUIRE, W.L. (1977). Estrogen receptor as an independent prognostic factor for early recurrence in breast cancer. *Cancer Res.*, **37**, 4669.

LEAKE, R.E., LAINNG, L., McARDLE, C. & SMITH, D.C. (1981). Soluble and nuclear oestrogen receptor status in human breast cancer in relation to prognosis. *Br. J. Cancer*, **43**, 6.

LIPPMAN, M.E. & ALLEGRA, J.C. (1980). Quantitative estrogen receptor analyses: the response to endocrine and cytotoxic chemotherapy in human breast cancer and the disease-free interval. *Cancer*, **46**, 2829.

OSBORNE, C.K., YOCHMOWITZ, M.G., KNIGHT, III, W.A. & McGUIRE, W.L. (1980). The value of estrogen and progesterone receptors in the treatment of breast cancer. *Cancer*, **46**, 288.

PETO, R., PIKE, M.C., ARMITAGE, P. & 7 others. (1977). Design and analysis of randomized clinical trials requiring prolonged observation of each patients. *Br. J. Cancer*, **35**, 1.

SAEZ, S., PICHON, M.F., CHEIX, F. & 4 others. (1983). Progesterone receptors and prognosis in early breast cancer: the experience of two centers. In: *Progesterone and Progestins*. (Eds. Bardin *et al.*), New York: Raven Press, p. 355.

SCATCHARD, G. (1949). The attraction of proteins for small molecules and ions. *Ann. N.Y. Acad. Sci.*, **51**, 660.

WESTERBERG, H., GUSTAFSON, S.A., NORDENSKJÖLD, B., SILFVERSWÄRD, C. & WALLGREN, A. (1980). Estrogen receptor level and other factors in early recurrence of breast cancer. *Int. J. Cancer*, **26**, 429.

VIHKO, R., JÄNNE, O., KONTULA, K. & SYRJÄLÄ, P. (1980). Female sex steroid receptor status in primary and metastatic breast carcinoma and its relationship to serum steroid and peptide hormone levels. *Int. J. Cancer*, **26**, 13.